# Vibration-induced injuries in workers exposed to transient and high frequency vibrations

**DOI:** 10.1186/s12995-020-00269-w

**Published:** 2020-06-17

**Authors:** Lars Gerhardsson, Christina Ahlstrand, Per Ersson, Ewa Gustafsson

**Affiliations:** Department of Occupational and Environmental Medicine, School of Public Health and Community Medicine, Institute of Medicine, Sahlgrenska Academy, University of Gothenburg, Medicinaregatan 16A, Box 414, SE-405 30 Gothenburg, Sweden

**Keywords:** Neurophysiologic effects, Vibration white fingers, Musculoskeletal disorders, Work ability

## Abstract

**Background:**

The risk of developing vibration white fingers and neurosensory symptoms increases with the duration and intensity of the exposure. The aim of this study was to investigate the risk of developing vibration white fingers (VWF), neurosensory symptoms and musculoskeletal disorders among workers exposed to transient and high frequency vibrations.

**Methods:**

The study included 38 vibration exposed workers from a loader assembly plant in Sweden (30 males and 8 females). All participants answered questionnaires and had a structured interview about work and medical history. A following medical examination included the determination of vibration and temperature perception thresholds and musculoskeletal symptoms in the neck, shoulder, elbow and hands. The individual vibration exposure expressed as A (8)-values and vibration exposure in minutes per day, were obtained from questionnaires answered by the participants.

**Results:**

The prevalence of VWF was 30% among the male workers and 50% among the females. The corresponding prevalence of neurosensory symptoms was 70% among the males and 88% among the females. Musculoskeletal findings were common among the male workers. Dominant symptoms/syndromes were tension neck syndrome, biceps tendinitis, carpal tunnel syndrome and ulnar entrapment in hand/wrist. A total of 32 diagnoses were observed among the male workers and four diagnoses among the female workers. Numbness in fingers and age had the strongest impact on perceived work ability.

**Conclusions:**

ISO 5349-1 considerably underestimates the risks of VWF for this group of workers exposed to transient and high frequency vibrations. It is therefore important to develop a risk assessment standard also covering this frequency range.

## Introduction

Long-term vibration exposure may lead to hand-arm vibration syndrome (HAVS). Dominant symptoms are vibration white fingers (VWF) and neurosensory symptoms and signs in the hands, such as numbness, tingling, reduced grip strength and decreased manual dexterity [1] The reason for developing either vascular or neurosensory symptoms or both is not yet clarified.

The risk of developing HAVS increases with the duration and intensity of the exposure [2]. The individual susceptibility varies. Sensitive subjects may develop quite severe symptoms within just a few years of exposure, while other subjects can work for decades without other than minor symptoms.

Vibration exposure causes vasoconstriction in the arteries in hands and fingers, smooth muscle wall hypertrophy, periarterial fibrosis and damage to endothelial cells and receptors. The damage to the endothelial wall is followed by platelet aggregation, release of serotonin and thromboxane as well as of epinephrine, nor-epinephrine and endothelin-1. The concentrations of vasoconstrictors such as nitrous oxide and prostacyclin are lowered [2].

In the nerves, tissue edema and vasospasm from vibration exposure may cause sensory loss based on nerve demyelination, axonal atrophy and degeneration of cell bodies as well as fibrosis and proliferation of Schwann cells [2].

The perception of vibration exposure is mediated by mechanoreceptors in the skin, e.g. Meissner’s and Pacinian corpuscles. Information from these mechanoreceptors is passing through large myelinated A-β fibres. Vibration exposure can also damage small myelinated A-δ fibres and unmyelinated C-fibres, which transmits temperature information about cold and warmth. Thus, both temperature and vibration perception thresholds can be used to assess neurosensory symptoms and signs in exposed workers.

In total, about 400,000 Swedish workers have a vibration exposure exceeding 2 h per working day. For workers with an advanced stage of HAVS, there is often great personal suffering with difficulties to use their hands. A substantial proportion of these workers have been forced to quit their work because of serious vibration-related symptoms. The costs for the individual worker, the company and the society are significant.

A higher level of upper extremity disorders in vibration exposed workers have been reported by several authors [3, 4]. A great number of subjects with musculoskeletal pain in neck and shoulders have been noted after occupational exposure to vibration [5]. The HECO-method (Health Surveillance in Adverse Ergonomics Conditions) with a linked reference material has proved suitable for this type of investigations [6, 7]. Both HAVS and musculoskeletal symptoms can affect work ability, often defined as balance between work demands and personal resources [8, 9].

Several strategies have been used to reduce the risk of developing HAVS, e.g. information, work rotation, maintenance of the tools, ergonomically designed tool handles and use of anti-vibration gloves. None of them have proved successful in the long run. The number of patients referred to departments of Occupational and Environmental Medicine in Sweden for investigations of vibration related symptoms has not decreased during the last decade.

The ISO 5349-1 standard describes the general requirements for the measurement and evaluation of human exposure to hand-transmitted vibration. It covers frequencies up to 1250 Hz but several research initiatives indicate that high frequency and transient vibrations higher than 1250 Hz may be even more damaging to the vascular and nerve systems in the hands [10–12]. It is therefore important to investigate vibration related symptoms and signs in workers with exposure to high frequency vibrations, not covered by the standard ISO 5349-1.

### Aims

The aim of this study was to investigate the risk of developing VWF, neurosensory symptoms and musculoskeletal disorders among workers exposed to transient and high frequency vibrations.

## Material and methods

The present study is part of a large ongoing Swedish research project named Zero vibration injuries (30 partners), with the intention to achieve a considerable reduction of the vibration exposure from high-frequency tools. The study was approved by the ethical committee at the University of Gothenburg.

### Study population

The study included 38 vibration-exposed workers from the so-called medium line at a wheel loader assembly plant in the middle of Sweden (30 males and 8 females). The total number of workers at the medium line was about 70. The selection process of the participants was done in cooperation with the chief safety representative at the plant, and was also dependent on the shift work schedule. The mean-age among the male workers was 39.3 ± 10.3 yrs. and the mean-exposure time was 13.9 ± 7.3 yrs. The corresponding figures among the female workers were mean-age 36.8 ± 8.8 yrs. and mean exposure time 10.6 ± 6.3 yrs.

All participants signed a written consent and completed a basic questionnaire with questions about e.g. work and medical history, exposure time, type of vibrating tools and hand-arm vibration symptoms.

### Vibration exposure

The assembly line is divided into several workstations. Every subject is assigned to two or more workstations and they change workstation every 4 h. The work at the assembly line covers a large number of work-tasks and the vibration exposure differs between the workstations. The most commonly used tools are impact wrenches and anvils. The estimated median vibration A (8) exposure is 2.2 m/s^2^ (range 0.4–4.9 m/s^2^) for the right hand and 1.8 m/s^2^ (range 0.3–5.3 m/s^2^) for left hand.

### Medical examinations

A medical examination was performed by an experienced physician including 2-PD test, Semmes-Weinstein’s monofilament test (5 filament kit), sensitivity to pain (needle) and test of hand grip strength (Jamar). Neurosensory tests included the determination of thermal (TPT) and vibration perception thresholds (VPT), and were performed by a biomedical analyst. The symptoms and signs were staged according to the Stockholm Workshop Scale (SWS). The staging of the vascular syndrome goes from 0 to 4 and for neurosensory symptoms from 0 SN to 3 SN. The higher staging, the more serious is the disease. The participants were told to avoid vibration exposure during the day of the measurement as well as intake of tobacco and coffee at least 1 h before the medical tests.

A musculoskeletal examination of the neck, shoulders, arms and hands was performed by an experienced physiotherapist.

### Thermal thresholds

Thermal perception thresholds were determined using a unidirectional stimulation technique that is based on a commercially available test instrument with a Peltier element-based thermode of 25 × 50 mm (Termotest®; Somedic Sales AB). The pulps of digits 2 and 5 in both hands were tested with a starting temperature of 32 degrees for both cold and warmth. To facilitate the testing, the forearm and the wrist of the participant was supported. The perception thresholds to non-painful cold and warmth, respectively, were obtained by delivering six cold stimuli, followed by six warm stimuli in random order, at a rate of 1 °C/sec. At the first sensation of cold and warmth, the subject was instructed to press the button of a handheld switch. The temperature then decreased or increased by 1 °C per second until the subject released the response button. The test procedure was repeated another five times. The mean of the last four assessments for cold and warmth on the finger pulps was registered as the cold or warmth perception thresholds.

### Vibrotactile measurements

Sinusoidal vibrations were delivered to the pulps of digits 2 and 5 in both hands by the ascending-descending method of limits. The subject’s response was received by the VibroSense Meter® system (Vibrosense Dynamics, Malmö, Sweden). The testing included sinusoidal frequencies at seven frequencies (8 Hz, 16 Hz, 32 Hz, 64 Hz, 128 Hz, 256 Hz, and 512 Hz) all transmitted to the finger through a vibration probe with a diameter of 4 mm. The finger temperature had to be at least + 28 degrees before the test started and the contact force between the probe and the finger was 1 N. To make the testing more comfortable for the subject, the wrist and the forearm of the participant was supported. The magnitude of the vibration was increased until the patient could feel the vibration in the finger. When the participant pressed the response button, the vibration magnitude decreased until the subject released the response button. Then the vibration amplitude started to rise again. The rate of change of the vibration amplitude was 3 dB/s and for each frequency there were six reversals. Thereafter, the testing automatically continued to the next frequency.

All results were age-corrected [13, 14] by comparison with values from a reference population supplied by the manufacturer of the device. All participants used ear protective devices to exclude the noise from outdoor and indoor sources. A sensibility index (SI) was calculated by dividing the area under the curve from the patient with the corresponding area for the reference population, which was supplied by the manufacturer of the instrument. An SI-index < 0.8 is interpreted as an abnormal response. An excellent reliability has been observed in VPT-determinations in patients with diabetic neuropathy, ICC > 0.94 [15].

In this study, the equipment used (VibroSense Meter® system) for VPT determinations on dig 2 and 5 bilaterally covers 7 frequencies. It meets the standard ISO 13091-1 for measurements of vibration thresholds transmitted by mechanoreceptors and A-β fibres. Additionally, we have used the Termotest from Somedic Sales AB to monitor the temperature perception thresholds (TPT) in dig 2 and 5 bilaterally, which is related to the response in small myelinated and non myelinated nerve fibres. Such TPT measurements are not described in the standard ISO 13091-1. The company, Somedic Sales AB, has for many years been known for production of high quality instruments for Quantitative Sensory Testing (QST). By determining VPT as well as TPT it is possible to monitor vibration injuries in both small and large nerve fibres, thereby increasing the possibility of diagnosing neurosensory injuries.

### Hand grip force

A Baseline® Hydraulic Hand Dynamometer (Fabrication Enterprises Incorporated, New York, NY, USA) in position number 2 was used to estimate the hand grip force. The mean of three measurements was used as the hand grip strength in the right and left hand, respectively.

### Measurements of musculoskeletal symptoms and diagnosis

A clinical examination of musculoskeletal symptoms in neck, shoulders, elbows, and hands was performed according to the HECO-protocol (Health Surveillance in Adverse Ergonomics Conditions, MEBA in Swedish). The protocol contains separate sections for the two anatomical regions neck/shoulders and elbow/hands.

The examination consisted of questions about symptoms, tests of range of movements, tenderness at palpation, muscle strength, sensibility, and pain or tingling at specific provocations of joints, tendons, muscles or nerves. From the protocol, the prevalence of perceived symptoms during the past 7 days, and specific diagnoses for the separate anatomical regions were established from predefined criteria [7].

### Work ability

The concept of work ability is complex and multidimensional. Work ability is often described as the balance between a person’s resources and work demands. These factors are not static but may change during different periods during working life [8]. From this concept, the instrument work ability index (WAI) was developed and validated in Finland in the 1980s. The instrument covers seven dimensions about e.g. work demands, the worker’s health status and resources and is well used in clinical occupational health and research to assess work ability during health examinations and workplace surveys [16].

In our study, work ability was measured by using the first item named work ability score (WAS) from the WAI questionnaire. It is a self-assessment of current work ability level compared to life-time best and has shown a strong association and an equally good predicted value with regard to sick leave, health and reported pain as the whole WAI-instrument [17, 18].

### Measurements of exposure

The exposure estimation was based on the information provided by the participants in the questionnaires including time of exposure to vibrating tools (months/years, daily exposure time in minutes), work tasks, type of vibrating tools, percentage use of the right hand, left hand or both.

Information about the type of tools used at each workstation was obtained from the company’s line power tool inventory list. The inventory list also contained information on the tools’ vibration levels. Technicians from the Research Institute of Sweden (RISE) made additional on-site vibration level measurements.

The combined data from the questionnaires, the additional measurements at the plant and the inventory list formed the basis for the individual vibration exposure estimations.

The average tool vibrations (ISO-values) for the entire production line were as follows: Pneumatic impact wrenches 5.4 m/s^2^, battery powered impact wrenches 10 m/s^2^, wrench 13 m/s^2^ and screw drivers 2.8 m/s^2^. The median vibration exposure among the participants was estimated to 2.2 m/s^2^ in the right hand and 1.8 m/s^2^ in the left hand.

### Statistics

Normal probability plots and Levene’s test were used to test the normality of the input variables. As the majority of the variables investigated showed a skewed distribution, non-parametric statistics was used for the statistical calculations.

Differences between groups were evaluated with the Mann-Whitney U-test. The relationship between variables was investigated by calculating the Spearman rank order correlation coefficients. *P*-values < 0.05 were regarded as statistically significant.

A multiple regression analysis was performed to elucidate which individual and work related factors that was most closely related to the workers’ work ability score (WAS).

All calculations were performed with the Statistical Package for the Social Sciences [19].

## Results

### Neurosensory findings

The prevalence of pathologic test results for 2-PD, monofilament and the needle test varied for each item from 5 to 16% (Table 1). The outcome was fairly similar for these three tests.

**Table 1:** Test results for 2-PD, monofilament and needle tests

2-PD	Pathologic	Monofilament	Pathologic	Needle test	Pathologic
Dig 2 dx	2	Dig 2 dx	6	Dig 2 dx	6
Dig 5 dx	6	Dig 5 dx	3	Dig 5 dx	6
Dig 2 sin	2	Dig 2 sin	5	Dig 2 sin	6
Dig 5 sin	3	Dig 5 sin	3	Dig 5 sin	5

Test results for 2-PD and Semmes Weinsteins’s monofilament (5 filament kit) tests, respectively, in dig 2 and 5 in right and left hand of 38 vibration exposed workers. Normal reference limit for 2-PD is ≤5 mm and for the monofilament test ≤3.61. Pain-test was performed with a sharp needle. The response was graded as normal or pathologic (feeling superficial touch but no sharpness). Dx = dexter = right, Sin = sinister = left. Dig 2 = digit 2 = index finger; dig 5 = digit 5 = little finger. Pathologic = number of pathologic test results.

The prevalence of pathologic test results for TPTs varied for each location between 16 and 37% (Table 2). The corresponding findings for VPT measurements in dig 2 and 5 bilaterally were 18% and for hand grip force (Jamar), 28% bilaterally. The most deviating findings among these six tests were thus noted for temperature perception thresholds and hand grip strength.

**Table 2:** Test results for TPT

Termotest	Pathologic
Dig 2 dx C	8
Dig 5 dx C	14
Dig 2 sin C	7
Dig 5 sin C	9
Dig 2 dx W	8
Dig5 dx W	12
Dig 2 sin W	6
Dig 5 sin W	6

Temperature perception thresholds (TPTs) in dig 2 and 5 in right and left hand. The reference interval is between 23 and 42 °C for subjects < 45 y and between 20 and 45 °C for subjects ≥45 y. C = cold and W = warmth. Dx = dexter = right, Sin = sinister = left. Dig 2 = digitus 2 = index finger; dig 5 = digitus 5 = little finger. Pathologic = number of pathologic test results.

The workers in this study show more serious neurosensory findings than vascular symptoms (Table 3). Four male workers were staged as SWS 3 SN, five male and female workers as SWS 2 SN and 19 workers as SWS 1 SN, giving a total prevalence of pathologic neurosensory findings of 74% in the right hand (70% in males and 88% in females). The prevalence for VWF was 30% in male workers and 50% in female workers.

In the left hand, the figures were very similar with a 68% total prevalence of pathologic neurosensory findings (males 63%, females 88%). The figures for VWF were the same as for the right hand (males 30%, females 50%).

**Table 3:** Grading of vibration white fingers and neurosensory findings

SWS RH	All w	Males	Females	SWS LH	All w	Males	Females
SWS 0	25	21	4	SWS 0	25	21	4
SWS 1	3	2	1	SWS 1	3	2	1
SWS 2	10	7	3	SWS 2	10	7	3
SWS 3	0	0	0	SWS 3	0	0	0
SWS 0 SN	10	9	1	SWS 0 SN	12	11	1
SWS 1 SN	19	13	6	SWS 1 SN	17	11	6
SWS 2 SN	5	4	1	SWS 2 SN	5	4	1
SWS 3 SN	4	4	0	SWS 3 SN	4	4	0

Grading of VWF and neurosensory findings (SN) in the right (RH) and left hand (LH) according to the Stockholm Workshop Scale (SWS). All w = all workers.

Age, exposure time, number of phalanges with Raynaud’s phenomenon and neurosensory symptoms in fingers as well as latency time for vascular and neurosensory findings did however, not differ significantly between male and female workers. As could be expected the male workers performed significantly better on the hand grip strength tests.

The mean latency time for VWF was 10 yrs. (median 8 yrs.; range 1–23 yrs) for male workers and 5 yrs. (median 5 yrs., range 1–8 yrs) for female workers. For neurosensory findings (numbness) the mean latency time for male workers was 6 yrs. (median 4 yrs., range 1–16 yrs) and 6 yrs. (median 7 yrs., range 1–8 yrs) for female workers.

The number of phalanges with numbness in the right hand did correlate fairly well with the number of phalanges with vibration white fingers (*r*_s_ = 0.50; *p* = 0.001) in the right hand as well as with the right-hand grip force (*r*_s_ = − 0.35; *p* = 0.033).

### Musculoskeletal diagnoses

Musculoskeletal findings were common among the male workers (Table 4). Dominant symptoms/syndromes were tension neck syndrome, biceps tendinitis, carpal tunnel syndrome and ulnar entrapment in hand/wrist. A total of 32 diagnoses were found among the male workers and four diagnoses among the female workers.

**Table 4:** Prevalence of musculoskeletal diagnoses

Site and syndrome	Male workers (*N* = 30)	Female workers (*N* = 8)
Tension neck syndrome	4	2
Thoracic outlet syndrome	1	1
Acromioclavicular syndrome	1	–
Biceps tendinitis	4	–
Supraspinatus tendinitis	3	–
Infraspinatus tendinitis	3	–
Lateral epicondylitis	1	–
Overused hand syndrome	1	–
Radial tunnel syndrome	2	–
Ulnar entrapment in elbow	3	–
Carpal tunnel syndrome	4	1
Ulnar entrapment in hand/wrist	5	–

Number of musculoskeletal diagnoses in neck/shoulders and elbow/hands among male and female workers. - = zero cases.

### Prevalence of VWF and neurosensory findings in relation to other studies

When using the workers’ own exposure time estimations, a 10% prevalence of VWF would be expected after 14 years of exposure according to the ISO 5349-1 standard. Calculations based on a systematic review and meta-analysis by Nilsson et al. [20] would yield a 10% prevalence after 15 years of exposure. In our study, male workers showed a prevalence of 30% after a mean exposure of 14 yrs. and females a prevalence of 50% after a mean exposure of 11 yrs. (Table 3).

The prevalence of neurosensory findings was 70% among male workers after a mean exposure of 14 yrs., and 88% among female workers after a mean exposure of 11 yrs. According to the meta-analysis by Nilsson et al. [20], a 10% prevalence would be expected after 5 yrs.

### Work ability

A multiple regression analysis was undertaken to study which variables that showed the strongest association with the independent variable Work Ability Score (WAS – stage 1 to 10) in the right hand. Two variables were included in the final model with the following equation:

Current estimated work ability (WAS) = 12.22–0.09 x age – 0.23 x number of phalanges with numbness in the right hand (multiple *r* = 0.64, *p* < 0.001). The corresponding 95% confidence intervals were as follows: age (− 0.14; − 0.04); number of phalanges with numbness in the right hand (− 0.35; − 0.11). Numbness gave the strongest unique contribution to the model (Beta − 0.53) followed by age (Beta − 0.49).

The corresponding outcome in the left hand was very similar with the following equation:Current estimated work ability (WAS) = 11.09–0.07 x age – 0.14 x number of phalanges with numbness in the left hand (multiple *r* = 0.50, *p* = 0.006). The 95% confidence intervals for the explanatory variables were (− 0.12; − 0.02) for age and (− 0.26; − 0.02) for number of phalanges with numbness in the left hand. In this case age gave the strongest contribution to the model (Beta − 0.39) followed by numbness (Beta − 0.35).

Age and exposure-time were closely related with a *r*_s_ = 0.62 (*p* < 0.001) in males and a *r*_s_ = 0.76 in female workers (*p* < 0.029). This is probably the reason why exposure time to vibrating tools was not included in the multivariate model.

## Discussion

In this study about 1/3 of the workers had developed vibration white fingers and about 75% neurosensory findings even though the estimated A (8)-median values were not higher than 2.2 and 1.8 m/s^2^, in the right and left hand, respectively. Both values were clearly below the current action limit value of 2.5 m/s^2^. The A (8)-value, however, is not a reliable measure of the vibration exposure as it only covers frequencies up to 1250 Hz. It is neither a good predictor of the development of HAVS for workers exposed to transient and high frequency vibrations, e.g. from impact or high speed rotary tools.

According to the Stockholm Workshop Scale, 13 workers were classified as VWF (3 subjects had stage 1 and 10 had stage 2), while 25 subjects had no symptoms in right hand (Table 3). Twenty-eight workers showed neurosensory symptoms and signs (19 stage 1 SN, 5 stage 2 SN and 4 stage 3 SN) in right hand. Ten workers had no symptoms.

The vasoconstriction in the fingers are dependent on the frequency of vibration [21]. HAVS can seriously affect both work-related activities and daily life. Two of the activities most affected include getting dressed and lawn maintenance [22]. During cooler weather periods gloves are often used, sometimes anti-vibration gloves. Even if the latter are ineffective as to the dampening of the hand-arm vibrations, they keep the worker’s hands dry and warm and protect them from abrasions, cuts, burns, chemical and biological exposures, thus having a preventive affect against HAVS [23].

A recent review and meta-analysis of risks for vascular and neurological diseases following hand-arm vibration exposure has been published by Nilsson et al. [20]. An A (8) exposure of 2.2 m/s^2^ (right hand) would lead to a 10% prevalence of white fingers after 14–15 years according to both the ISO-5349-1 standard and the meta-analysis by Nilsson et al. [20]. In our study, we found a prevalence of 30% (9/30) after a mean exposure time of 14 yrs. in males and a prevalence of 50% among females (4/8) after a mean exposure of 11 yrs.

A 10% prevalence of neurosensory findings in male workers following an A (8)-median exposure of 2.2 m/s^2^ will according to the meta-analysis by Nilsson et al. [20] be reached after 5 years of exposure. In our study, the prevalence was 70% in males after a mean exposure time of 14 y and 88% in females after a mean exposure of 11 y.

It has been suggested that cold thresholds are more sensitive indicators of early neurosensory disorders than warm thresholds [24]. However, no statistical difference between the neurosensory effect on cold and warm thresholds was found in our study.

Also, other types of high-frequency vibration may give similar vascular and neurosensory symptoms. For example, dentists and dental technicians are exposed to high frequencies [25] and levels around or higher than 6000 Hz are common. A vibration exposure with a frequency content mainly above 1250 Hz are not covered by the ISO 5349-1 standard. These high frequency components may damage the arteries and nerves in the hand of the worker [26]. Accordingly, the health risks of being exposed to transient and high frequency vibrations cannot be evaluated when measuring vibration according to the ISO-5349-1 standard [26].

The risk of obtaining Raynaud’s phenomenon has been studied by Engstrom and Dandanell [27] in the aircraft industry in Sweden. They investigated 340 riveters working in the aircraft industry who were exposed to high frequency and transient vibrations from riveting hammers, bucking bars, drills and rivet shavers. The employment time varied from 1 to 44 years. The exposure to riveting tools, which was the dominant exposure source to vibration, was no longer than 1 min per day. The total exposure to vibrating tools was 40 min per day. For the riveting hammer the vibration frequency content was between 1000 Hz and 10,000 Hz with a weighted acceleration according to ISO 5349-1 of 10 m/s^2^. Eighty-six of the workers were diagnosed with Raynaud’s syndrome (latency time 0–27 yrs.; median latency-time 11 yrs). When using 25 years of exposure as an endpoint, 288 workers were at risk. Of them 59 developed vibration white fingers, which could be compared with an expected number of 14 when using the ISO-5349-1 standard. This is a four-fold increase. Of workers exposed for more than 10 years, more than 50% developed Raynaud’s syndrome [11]. The authors conclude that the increased risk of developing Raynaud’s syndrome may be due to the exposure to impact and shock-wave accelerations. An increased risk of developing HAVS has also been reported by Barregard et al. [12] in a study of 806 car mechanics. The mean daily exposure to impact wrenches was 14 min/day and the mean duration of exposure was 12 yrs. About one quarter of the working force reported vibration white fingers after 20 years of exposure. The estimated figure calculated according to the ISO-5349-1 standard is about 3%. That is about eight times lower than the observed figure and a clear underestimation of the calculated risk assessment. The observed figure for neurosensory findings after 20 years of exposure was even higher (40%).

Two mechanisms that may explain the increased risk of developing HAVS by exposure to high frequency vibrations have been discussed by Starck [10]. High impact vibrations may distort the tissues in the hand and damage the endothelium in the arteries, which may lead to erythema and swelling of the fingers after exposure. As impulsive vibration spreads to larger areas in finger and hands, more receptors are activated through temporal and spatial summation, which causes increased neurosensory symptoms and signs [10]. The impulse character of the vibration can increase the risk of developing HAVS. High impact components were observed as being the most hazardous in a study of stone workers as their highly impulsive vibration exposure caused a considerably higher number of subjects with vibration white fingers than would be expected according to the ISO 5349-1 standard [28] .

In a 3-year follow-up study of 249 hand-arm vibration exposed workers and 138 referents, the workers had a higher prevalence and cumulative incidence of neurosensory disorders in hands and a reduced work ability compared to the referents [29]. The intensity of the daily vibration exposure and the duration of exposure in years were significantly related to both neurosensory findings and work ability [29]. The vibration perception threshold was the most sensitive QST-parameter to diagnose adverse health neurosensory findings in a study of chain saw workers and stone cutters [30]. Numbness in fingers was a prominent symptom in workers with HAVS.

In a Swedish study of 30 dentists and 30 dental technicians exposed to high frequency tools, Akesson et al. [31] observed typical neurosensory symptoms in both groups. A significant impairment of vibrotactile sensibility, strength and motor performance was noted, although their exposure to vibration was very low according to the ISO 5349-1 standard.

The results from the clinical examination in our study showed a high prevalence of musculoskeletal symptoms and diagnoses in neck, shoulders, elbows and wrist/hands in the study group. This finding can be explained by the ergonomic exposure at the assembly line, i.e. repetitive movements with arms and hands, forceful grips, and non-neutral postures in neck, shoulders and wrists. In a study of riveters in the aircraft industry in the Netherlands, Burdorf and Monster [32] found that use of impact power tools can increase the risk of developing pain and stiffness in hands, wrist, arms and shoulders. The high prevalence of vibration related symptoms, however, cannot be explained by these factors.

In our study, a multiple regression analysis was undertaken with WAS (work ability score) [17, 33] as the dependent variable. The analysis showed that the explanatory variables age and number of phalanges with numbness gave a significant impact on the model (R^2^ = 0.41 in the right hand and R^2^ = 0.25 in the left hand). As most workers were right-handed, the right hand probably had the highest exposure to vibrating tools, which may explain the higher R^2^ value. Numbness in fingers gave the strongest contribution to the model in the right hand, followed by age. Age and exposure time were closely associated, which explains why exposure-time was not included in the model.

Several factors may influence the work ability of a subject, e.g. humidity, high temperature, dust, noise, vibration, ergonomic risk factors, physical factors such as high work load, low job control and low social support [34–36]. The outside world and a subject’s social life also has great significance.

### Limitations

The total number of workers at the medium line was around 70, and 38 of them (54%) were included in the study group. The selection process of the participants was done in cooperation with the chief safety representative at the plant, and was also dependent on the shift work schedule. This process may have influenced the results. Although, this may be the case, the observed figures for VWF and neurosensory findings are alarming. Another limitation is the small number of female workers, which hampers the interpretation of their results, and the comparison with the male workers at the plant.

### Summary

Several studies from the 1980’s and 1990’s indicate that transient and high frequency vibrations can damage nerves, arteries and corpuscles in hands and fingers. A considerably higher prevalence of VWF than would be calculated from the ISO-5349-1 standard has been observed in several studies [11, 12, 27]. The repeated stretching of skin, arteries, nerves and corpuscles and the shock wave that propagates into the fingers may not give the tissues enough time to recover. Such a damaging mechanism may not take place in exposures within the ISO 5349-1 range. This is probably one important explanation to the observed high prevalence of VWF and neurosensory findings in our study. Nerves are affected earlier than arteries and nerves are also causing significantly more serious symptoms and signs from a long-term perspective. As shown in our study, the observed prevalence of VWF by far exceeds the estimated figures that can be calculated from the ISO 5349-1 standard. Furthermore, considerably higher prevalence figures are observed for neurosensory findings, which however, are not covered by the ISO 5349-1 standard. A median A (8) vibration exposure of 2.2 and 1.8 m/s^2^, respectively, as found in our study, is below the action limit of 2.5 m/s^2^. With such a low to moderate exposure, vascular and/or neurosensory findings of this magnitude would not be expected. Instead, we have observed very high and alarming prevalence figures, especially for neurosensory symptoms. As previously reported in several animal experiments, shock wave exposure causes severe nerve damage [37–39]. Thus, both human and animal studies show that the ISO 5349-1 standard considerably underestimates the observed risks for VWF in subjects exposed to transient and high frequency vibration. It is therefore important to develop a risk assessment for vascular and neurosensory findings that includes both vibrations covered by the ISO 5349-1 standard and transient and high frequency vibrations above 1250 Hz.

## Conclusions

A high prevalence of VWF and neurosensory findings was observed in the present study despite the fact that exposure to frequency vibrations within the ISO 5349-1 standard range was clearly below the action limit (2.5 m/s^2^). The study group also had an exposure to high frequency vibrations (> 1250 Hz) that are not taken into account in the ISO 5349-1 standard. This is probably the main explanation for the high prevalence of observed vibration related symptoms and signs. Accordingly, an annex to the current ISO 5349-1 standard needs to be enforced, which also covers exposure to high frequency vibrations. It is also important that the company health service sector as well as employers, workers and manufacturers of tools producing transient and high frequency vibrations are informed about the high risk of developing HAVS when using this type of tools.

## Data Availability

The datasets used and/or analysed during the current study are available from the corresponding author on reasonable request.
